# Neutrophil-Derived Semaphorin 4D Induces Inflammatory Cytokine Production of Endothelial Cells via Different Plexin Receptors in Kawasaki Disease

**DOI:** 10.1155/2020/6663291

**Published:** 2020-12-16

**Authors:** Junhua Huang, Shouzhen Wu, Sancheng Cao, Xieying Zhu, Shuwan Zhang

**Affiliations:** ^1^School of Medical Technology, Xi'an Medical University, Xi'an, Shaanxi Province 710021, China; ^2^Department of Clinical Laboratory, The Affiliated Children's Hospital of Xi'an Jiaotong University, Xi'an, Shaanxi Province 710003, China

## Abstract

Inflammation of endothelial cells (ECs) plays an important role in the pathogenesis of coronary artery lesions (CALs) in Kawasaki disease (KD). Semaphorin 4D (Sema4D) is the first semaphorin shown to have immunoregulatory functions by interacting with its receptors—plexin Bs. Recently, Sema4D has been reported to exert a proinflammatory effect on the endothelium and to be involved in cardiovascular disease. However, the role of Sema4D in KD remains unknown. This study was aimed at revealing the change of soluble Sema4D (sSema4D) in the serum of patients with KD and the effect of the sSema4D-plexin axis on the production of proinflammatory cytokines from human coronary endothelial cells (HCAECs) stimulated with sera from KD patients. Our results showed that serum sSema4D levels were specifically elevated in KD patients, especially in those with CALs, and correlated positively with disease severity and serum concentrations of interleukin- (IL-) 1*β*, IL-6, and IL-8. The disintegrin and metalloproteinase domain 17- (AMAM17-) mediated Sema4D shedding from neutrophils contributed to the elevation of sSema4D in the serum of KD patients. Furthermore, we found that Sema4D induced IL-1*β* production of HCAECs via plexin B2, whereas it promoted IL-6 and IL-8 production via plexin B1. Moreover, the expression of both plexin B1 and plexin B2 was upregulated in HCAECs treated with KD sera, and silencing of the two plexin receptors suppressed the overexpression of IL-1*β*, IL-6, and IL-8 in KD serum-treated HCAECs. Thus, our findings indicated that sSema4D released from neutrophils participates in the pathogenesis of KD-CALs by promoting inflammatory cytokine production of ECs via both plexin B1 and plexin B2, and Sema4D may be a novel predictor for KD-CALs and a candidate therapeutic target for anti-inflammatory strategies of KD.

## 1. Introduction

Kawasaki disease (KD), a kind of systemic immune vasculitis with unknown etiology and unclear pathogenesis, is a leading cause of acquired heart disease in children under 5 years of age [1]. When KD occurred, systemic medium-sized vessels, especially coronary arteries, are primarily affected. If there is no timely medical intervention, such as intravenous immunoglobulin (IVIG) therapy, coronary artery lesions (CALs), such as coronary artery aneurysms (CAA), could occur in 20-30% of KD children and bring longer hospitalizations and higher hospital charge [2]. Although the exact pathological mechanism of KD-CALs still remains obscure, a lot of research evidence has shown that KD-CALs are closely associated with the overexpression and release of a variety of proinflammatory cytokines, such as interleukin- (IL-) 1*β*, IL-6, and IL-8 [3]. Furthermore, recent studies have indicated that inflammation of vascular endothelial cells (ECs) plays a pivotal role in the pathogenesis of CALs in KD [4, 5], and it is thought that ECs, as a source of proinflammatory cytokines [6], are stimulated to express and secret a variety of inflammatory factors in pathological status, which in turn cause recruitment and infiltration of inflammatory cells, thus resulting in subsequent local damage of vascular wall and formation of CAA [7]. However, the mechanisms by which ECs produce proinflammatory factors under KD condition remain largely unknown.

Semaphorin 4D (Sema4D), also called immune semaphorin, is a member of the class 4 semaphorins, which has been shown to be implicated in both immune regulation and inflammatory response, although it was firstly found to be essential for proper neuronal development [8, 9]. As a transmembrane protein, membrane-bound Sema4D (mSema4D) is mainly expressed on the surface of immune cells, such as T cells, B cells, neutrophils, monocytes/macrophages, and platelets. After cellular activation, mSema4D can be cleaved by several sheddase from the cell surface to yield its soluble form (called sSema4D), resulting in an increased level of sSema4D in the peripheral blood [10]. Indeed, increased sSema4D level in serum has been reported in many inflammatory immunopathogenesis diseases, such as rheumatic arthritis [11], antineutrophil cytoplasmic antibody-associated vasculitis [12], and coronary artery stenosis [13]. Notably, as a ligand, sSema4D exerts biological functions via interaction with its receptors, including plexin B1/B2 on nonlymphoid tissues and CD72 on lymphoid tissues [14]. By binding to different receptors, sSema4D exhibits different effects on the inflammatory phenotype in different types of cells. For instance, the Sema4D-plexin B1 axis promoted the inflammation of chondrocytes [15], whereas the Sema4D-plexin B2 axis contributed to the keratinocyte's inflammation [16]. Interestingly, studies have shown that both plexin B1 and plexin B2 are expressed on the surface of human ECs [17, 18]. Importantly, sSema4D has recently been reported to be an inducer of the production of some proinflammatory cytokines and to be involved in endothelium inflammation and vascular dysfunction [12, 19]. However, the proinflammatory effects of sSema4D on the endothelium have not been sufficiently clarified, and to our knowledge, the role of Sema4D in KD has not been investigated.

Therefore, based on the above-mentioned evidence, we hypothesize that Sema4D is involved in the pathogenesis of KD-CALs by inducing proinflammatory cytokine production of ECs.

## 2. Subjects and Methods

### 2.1. Human Subjects

Fifty patients with acute KD before IVIG therapy and 20 healthy children between January 2019 and December 2019 at the Affiliated Children's Hospital of Xi'an Jiaotong University (Xi'an, China) were enrolled in this study, and blood samples were collected from these individuals. Of the 50 KD patients, 31 underwent the second blood collection at the convalescent phase and 18 had CALs at the acute phase. All patients diagnosed with KD met the criteria of *Diagnosis, Treatment, and Long-Term Management of Kawasaki Disease* by the American Heart Association in 2017 [20]. To determine the presence of CAL, we detected the following 7 sites of the coronary artery by echocardiography: left main coronary artery (between its opening and the bifurcation of the circumflex branch), left anterior descending-proximal segment (3~5 mm after its opening), left circumflex branch (3~5 mm after its opening), right coronary artery- (RCA-) proximal segment (3~5 mm after its opening), RCA-middle segment (right atrioventricular groove), RCA-distal segment (right posterior atrioventricular groove), and posterior descending coronary artery (posterior interventricular groove). When the *Z*-score of one or more of the detected sites in these coronary arteries was ≥2, the KD patient was defined as having CAL (If the *Z*-score of more than one site presented ≥2, the largest one was recorded). This research was approved by the Ethics Committee of the Affiliated Children's Hospital of Xi'an Jiaotong University. Informed consent was obtained from the guardians of all participants included in this study.

### 2.2. Cell Culture and Preparation

Human coronary artery endothelial cells (HCAECs) were obtained from SCIENCELL (CA, USA) and cultured in P1640 medium with 10% fetal bovine serum (FBS). When HCAECs were 70-80% confluent, the cells were seeded into 6-well microplates. Subsequently, when HCAECs were 90% confluent, the cells were cultured in P1640 medium containing 20% KD sera or sera of healthy children for 6 hours at 37°C and 5% CO_2_ condition. For the recombinant Sema4D (rSema4D) stimulation assay, different concentrations of rSema4D (R&D Systems, USA) were added into the mediums when HCAECs were 90% confluent, and the culture supernatants were collected after 6 hours for ELISA. For a transfection assay, siRNA of plexin B1 and plexin B2 were designed and synthesized by Sangon (Shanghai, China), and the Lipofectamine 3000 reagent (Invitrogen, CA, USA) was used to transfect siRNA of plexin B into HCAECs following the manufacturer's instructions.

### 2.3. ELISA

The levels of IL-1*β*, IL-6, and IL-8 in human serum and cell culture supernatant and the levels of sSema4D and ADAM17 in human serum were measured using 2-step sandwich ELISA kits (EliKine™, Abbkine, Wuhan, China) according to the manufacturer's instructions. Serum samples were stored at -80°C until use and analyzed in duplicate.

### 2.4. Flow Cytometry (FCM)

Blood samples were prepared in sodium citrate anticoagulant tubes and then analyzed on NovoCyte D1040 (ACEA, USA) using NovoExpress software (ACEA, USA). The following antibodies were used: PE-conjugated anti-human Sema4D, FITC-conjugated anti-human CD45, PECY5-conjugated anti-human CD3, PECY5-conjugated anti-human CD19, PECY5-conjugated anti-human CD14, PECY5-conjugated anti-human CD15, and FITC-conjugated anti-human CD61. All antibodies were purchased from BioLegend (CA, USA).

### 2.5. Quantitative Real-Time PCR (qRT-PCR)

Total RNA from HCAECs was extracted by using the RNeasy Mini Kit (Qiagen, Germany) and then reverse-transcribed to complementary DNA by using the PrimeScript RT Mix (Takara, Dalian, China). The mRNA expression levels of plexin B1, plexin B2, IL-1*β*, IL-6, and IL-8 were analyzed using the SYBR Green Real-time PCR Mix (Takara, China) in the ABI 7500 analyzer (ABI, CA, USA).

### 2.6. Statistical Analysis

Quantitative data with a normal distribution were expressed as the mean ± standard deviation (*M* ± SD) and were compared using Student's *t*-test between two groups. The Pearson correlation analysis was performed to explore the relationship between serum sSema4D and inflammatory factors. A two-tailed *p* value < 0.05 was statistically significant. All statistical analyses were conducted using Prism 7.0 software (GraphPad, USA).

## 3. Results

### 3.1. sSema4D Levels Are Increased in the Serum of Patients with KD

**Figure 1:**
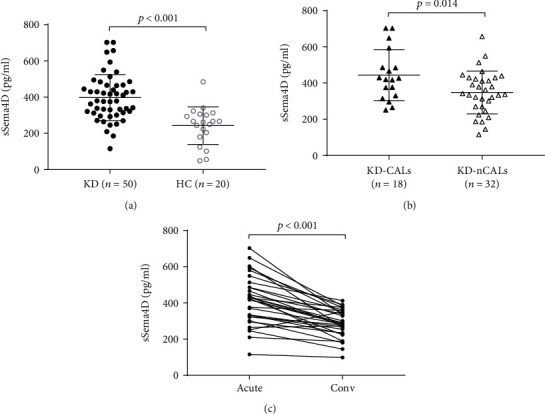
sSema4D levels are elevated in the serum of KD patients. (a) Levels of sSema4D in the serum from acute KD patients (KD) and healthy children (HC). (b) Levels of sSema4D in the serum from KD patients with CALs and without CALs. (c) The changes of serum sSema4D levels in the acute phase and convalescent phase of the same patient (*n* = 31). Values are *M* ± SD. The *p* value is determined by the unpaired *t*-test (a and b) and paired *t*-test (c).

To explore the pathological significance of Sema4D in KD, we first measured serum sSema4D levels in patients with KD by ELISA. We found that serum sSema4D levels were obviously higher in patients with KD than in healthy controls (Table 1 and Figure 1(a)). Moreover, serum levels of sSema4D in KD patients with CALs showed a significant elevation compared with the levels in those without CALs (Figure 1(b)). Notably, the serum sSema4D level in the convalescent phase of KD was clearly decreased compared with that in the acute phase (Figure 1(c)).

### 3.2. Serum sSema4D Levels Are Correlated with Disease Severity and Inflammatory Cytokine Concentrations

**Figure 2:**
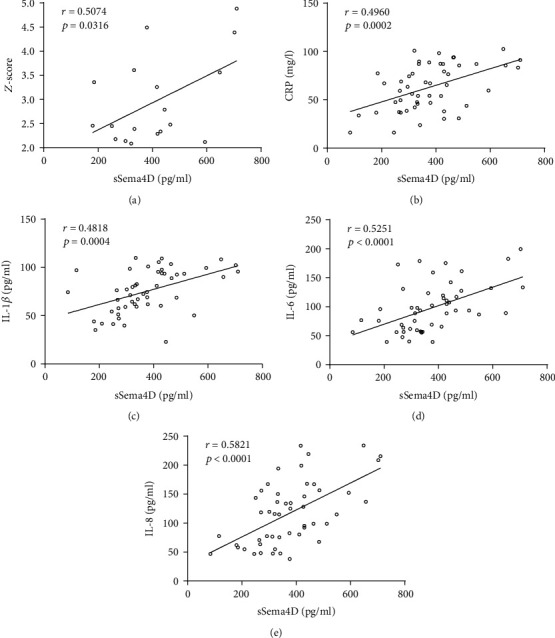
Elevated levels of serum sSema4D are positively correlated with disease severity and cytokine concentrations. (a, b) Correlation between serum sSema4D levels and *Z*-score (*n* = 18) and CRP (*n* = 50). (c–e) Correlations between serum sSema4D levels and concentrations of IL-1*β*, IL-6, and IL-8 in patients with KD (*n* = 50). Correlations are expressed as the Pearson correlation coefficient.

To investigate the clinical implications of sSema4D in KD, we examined the relationship between sSema4D levels and clinical parameters. The serum levels of sSema4D were positively correlated with the *Z*-score, an indicator of the CAL degree and disease severity, and CRP, a common inflammatory marker (Figures 2(a) and 2(b)). Previous studies have reported that Sema4D can induce IL-1*β*, IL-6, and IL-8 secretion [12, 16, 21], and these three cytokines have been proven to play important roles in the pathogenesis of KD [22–24]. So, we explored the association of sSema4D levels and concentrations of IL-1*β*, IL-6, and IL-8 in the serum of KD patients. As shown in Figures 2(c)–2(e), sSema4D levels are closely related to concentrations of these cytokines.

### 3.3. Neutrophils Contribute to the Elevation of Serum sSema4D in Patients with KD

**Figure 3:**
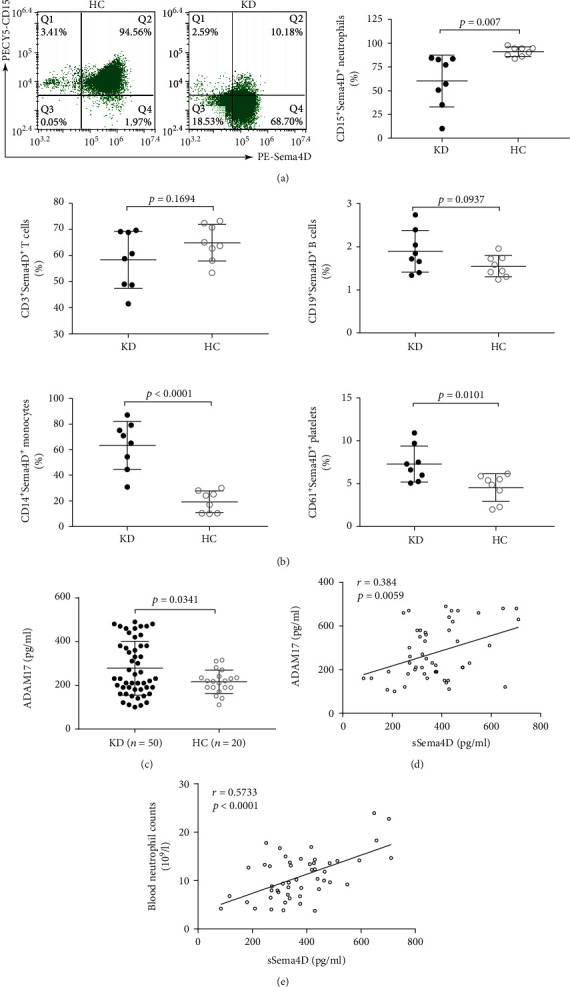
ADAM17-mediated Sema4D shedding in neutrophils is responsible for the high levels of sSema4D in KD serum. (a) Percentage of CD15^+^Sema4D^+^ neutrophils in CD15^+^ cells. The result shown is representative of FCM findings from KD patients (*n* = 8) and healthy children (*n* = 8). (b) Percentage of Sema4D^+^ cells in CD3^+^ T cells, CD19^+^ B cells, CD14^+^ monocytes, and CD61^+^ platelets from KD patients (*n* = 8) and healthy children (*n* = 8). (c) Elevated levels of serum ADAM17 in patients with KD. (d, e) Correlations between serum sSema4D levels and ADAM17 concentration (d) and neutrophil counts (e) in patients with KD (*n* = 50). Correlations are expressed as the Pearson correlation coefficient. KD: Kawasaki disease; HC: healthy children.

Next, to identify the cell source of elevated sSema4D in the serum of KD patients, we comprehensively analyzed the cell surface expression of mSema4D on peripheral blood cells using FCM. The results revealed that the mSema4D expression on CD15^+^ neutrophils, but not T cells, B cells, monocytes, or platelets, was lower than that of healthy children (Figures 3(a) and 3(b)). A recent study has shown that ADAM17 is the shedding proteinase of mSema4D in neutrophils [12]. To determine whether ADAM17 contributed to the mSema4D shedding on the neutrophil cell surface in KD condition, we measured the concentration of ADAM17 in the serum of KD patients. Consistent with our expectations, serum concentrations of ADAM17 were increased in KD patients (Figure 3(c)) and positively correlated with serum sSema4D levels (Figure 3(d)). Also, serum sSema4D levels were closely related to peripheral blood neutrophil counts (Figure 3(e)).

### 3.4. Sema4D Induces Inflammatory Cytokine Production of HCAECs by Interacting with Different Plexin Receptors

**Figure 4:**
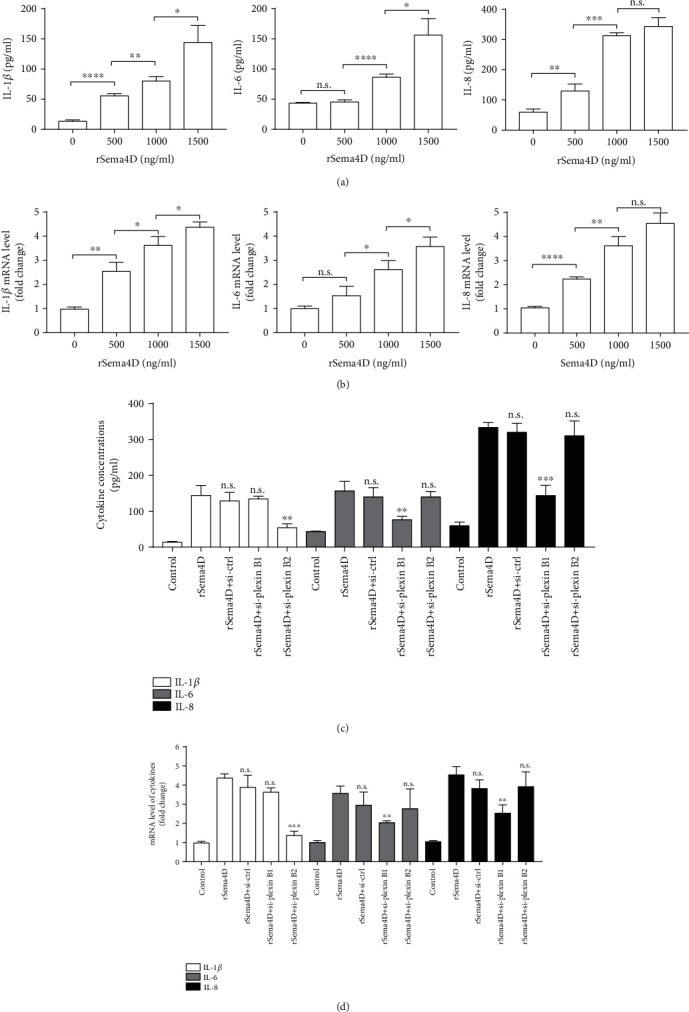
Sema4D induces IL-1*β*, IL-6, and IL-8 production of HCAECs via different plexin receptors. (a) Elevated concentrations of IL-1*β*, IL-6, and IL-8 in the culture supernatant of HCAECs stimulated with different doses of rSema4D. (b) Increased mRNA expression of IL-1*β*, IL-6, and IL-8 in rSema4D-stimulated HCAECs. ^∗^*p* < 0.05, ^∗∗^*p* < 0.01, ^∗∗∗^*p* < 0.001, and ^∗∗∗∗^*p* < 0.0001. (c, d) Decreased concentrations in the culture supernatant (c) and descent mRNA expression of IL-1*β*, IL-6, and IL-8 of HCAECs (d) after transfection with si-plexin B1 and si-plexin B2. rSema4D in (c) and (d) is 1500 ng/ml. ^∗∗^*p* < 0.01 and ^∗∗∗^*p* < 0.001 versus the rSema4D treatment group. n.s.: no significant difference with the rSema4D treatment group; control: serum-free HCAECs.

Inflammation of ECs plays a pivotal role in the pathogenesis of KD-CALs, and sSema4D exhibits proinflammatory effects on a variety of cells by inducing cytokine secretion. Here, to assess the proinflammatory role of sSema4D in KD, we investigated the effect of Sema4D on IL-1*β*, IL-6, and IL-8 production of endothelial cells. As shown in Figures 4(a) and 4(b)), ELISA and qPCR indicated that rSema4D promoted cytokine production of HCAECs in a dose-dependent manner. Furthermore, to identify the specific Sema4D-plexin B signaling pathway involved in the elevated expression of different cytokines, we utilized siRNA to silence plexin B1 and plexin B2 in HCAECs. We found that silencing plexin B1 only caused a significant decrease in IL-6 and IL-8 expression, whereas silencing of plexin B2 only suppressed the IL-1*β* expression of HCAECs stimulated with rSema4D (Figures 4(c) and 4(d)).

### 3.5. Plexin B1 and Plexin B2 Are Upregulated in HCAECs Treated with KD Sera

**Figure 5:**
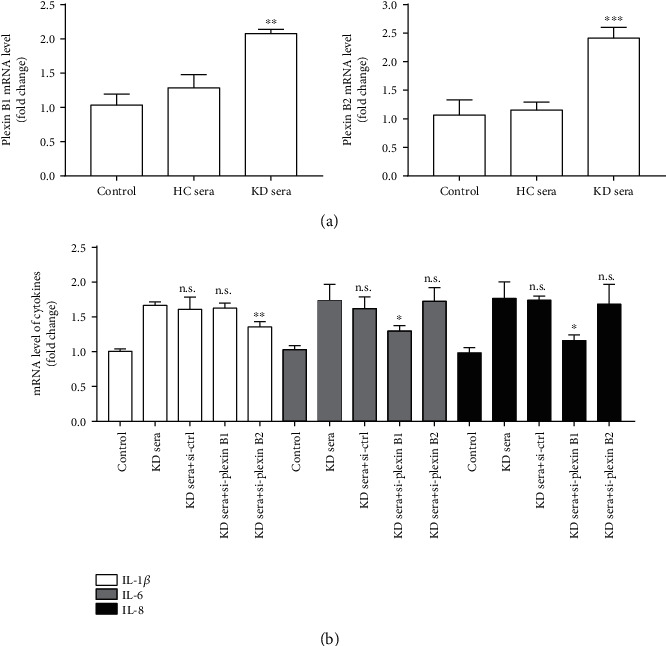
Sera from KD patients stimulated mRNA upregulation of plexin B1 and plexin B2 in HCAECs and silencing of plexin suppressed KD serum-induced overexpression of IL-1*β*, IL-6, and IL-8. (a) The mRNA expression levels of plexin B1 and plexin B2 in HCAECs treated with 20% KD sera (pooled from 15 patients) or 20% healthy control sera (HC sera) (pooled from 15 individuals). ^∗∗^*p* < 0.01 and ^∗∗∗^*p* < 0.001 versus the healthy control serum group. (b) The mRNA expression levels of IL-1*β*, IL-6, and IL-8 in HCAECs treated with 20% KD sera with or without si-plexin transfection. ^∗^*p* < 0.05 and ^∗∗^*p* < 0.01 versus the KD serum group. n.s.: no significant difference with the KD sera treatment group; control: serum-free HCAECs.

To further evaluate the effect of the Sema4D-plexin B axis on the expression of cytokines in KD, we first detected the expression of plexin B1 and plexin B2 in HCAECs treated with 20% KD sera (pooled from 15 patients). As shown in Figure 5(a), KD sera can stimulate HCAECs to upregulate the mRNA expression of plexin B1 and plexin B2. Furthermore, consistent with the findings in Section 3.4, silencing of the corresponding plexin receptor inhibited the KD serum-induced overexpression of IL-1*β*, IL-6, and IL-8 in HCAECs (Figure 5(b)).

## 4. Discussion

Inflammation of ECs is closely associated with the onset and progression of KD and its complication—CALs, which has not been fully illustrated [25]. Here, we identified a novel Sema4D-plexin B axis-mediated proinflammatory cytokine production mechanism of ECs and highlighted the pathological involvement of Sema4D in KD-CALs.

In this study, we found that serum sSema4D was significantly increased in KD patients compared with healthy controls, which is similar to the investigations of sSema4D in coronary heart disease [13] and autoimmune diseases [11]. KD is characterized by cardiovascular complications and the autoimmune process [26, 27], and both phenotypes are implicated in the inflammatory response. Thus, we deduced that elevation of circulating sSema4D may be a common phenomenon in inflammatory conditions, including KD. Indeed, we found that sSema4D levels were positively correlated with CRP concentrations, which was consistent with other studies [12, 13, 16, 19, 21, 28, 29] and further demonstrated the association of Sema4D and the inflammatory phenotype. Furthermore, sSema4D levels were much higher in KD patients with CALs than in those without CALs and correlated with the *Z*-score, which is similar to Gong et al.'s study that showed an increased level of serum sSema4D in coronary heart disease and a correlation between sSema4D levels and the extent of coronary artery stenosis [13], indicating that sSema4D in blood can reflect the severity of KD-CALs. Moreover, we showed an association of sSema4D and IL-1*β*, IL-6, and IL-8 (Figures 2(c), 2(d), and 2(e)), three of which play important roles in KD pathogenesis, suggesting that sSema4D may affect CRP levels by inducing proinflammatory cytokine production in KD patients. In addition, sSema4D showed a clear drop in KD patients with clinical remission. Similar changes were observed in patients with Hantaan virus infection [30] or rheumatoid arthritis [11]. Therefore, our findings suggest the possibility of sSema4D as a biomarker for predicting the severity of KD-CALs and monitoring the therapeutic effects of KD.

The source of circulating sSema4D has not yet been fully described under physiologic conditions, and which kind of blood cells contribute to its elevation in a pathological state remains to be elucidated. Accumulating evidence indicated that, in various diseases, sSema4D elevation in serum is often due to proteolytic cleavage of mSema4D from various blood cells, including neutrophils, lymphocytes, monocytes, and platelets by several metalloproteinases, such as ADAM17 [8]. Here, we comprehensively analyzed the cell surface presence of Sema4D in KD patients and found that the mSema4D expression on neutrophils, but not on other leukocytes or platelets, was downregulated in KD patients, suggesting that neutrophils were the main source of increased sSema4D in KD serum, which further verified the fact that the activation of the innate immune system appears more earlier than the adaptive immune activation in the pathological process of KD. A recent study showed that ADAM17 specifically mediated the shedding of neutrophil surface Sema4D, and the AMAM17 elevation in blood was essential for the shedding process [12]. Herein, similarly, we demonstrated that ADAM17 was increased in the serum of KD patients, which indicated that ADAM17 may contribute to the sSema4D elevation by proteolytically cleaving neutrophil mSema4D. Furthermore, we found that serum Sema4D levels were positively correlated with ADAM17 concentrations and blood neutrophil counts, further identifying that neutrophils contributed to the sSema4D elevation in KD via ADAM17-mediated Sema4D shedding. Interestingly, a relationship between ADAM17 gene polymorphism and KD has been reported [31]; therefore, ADAM17 may be involved in KD pathogenesis by one unknown mechanism, and our findings gave a clue. On the other hand, neutrophil surface Sema4D has been shown to act as a negative regulatory receptor of neutrophil activation, inhibiting the generation of reactive oxygen species (ROS) and the formation of neutrophil extracellular traps (NET) [12]. Intriguingly, recent studies reported that enhanced ROS production and NET release were importantly implicated in KD pathogenesis [32–34], while the mechanism remained, to a wide extent, unclear. Also, autopsy has suggested that neutrophil infiltration in the vascular wall is deeply involved in coronary artery damage in the early stage of KD [35]. Thus, we could propose a rational derivation that the reduction of mSema4D on neutrophils of KD patients not only means the sSema4D elevation in blood but also represents the abnormal neutrophil activation.

Several studies indicate that sSema4D, by bonding to its plexin receptor, functions as a promoter of proinflammatory cytokine production in various cells. For instance, Sema4D can induce IL-6 and TNF-*α* production of monocytes in rheumatic arthritis [11] and promote IL-6 secretion of nasal epithelial cells [21] and Sema4D-plexin B2 promoted the production of IL-1*β* and IL-18 by keratinocytes [16]. For the first time, we found that Sema4D can induce HCAECs to produce IL-1*β*, IL-6, and IL-8 via different plexin B receptors. Our finding was similar to the above-mentioned studies and further proved the proinflammatory role of Sema4D in a broad variety of cells. Of note, two studies have shown that Sema4D-plexinB1 can mediate IL-8 release in human umbilical vein endothelial cells (HUVECs) [12, 17], and our results were in line with their findings, suggesting that the Sema4D-plexin B1 axis-induced IL-8 production was existing in both vein ECs and artery ECs, despite the fact that HUVECs are different from HCAECs in some biological features [36]. Additionally, we showed that Sema4D can also mediate IL-1*β* and IL-6 release, respectively, by interacting with plexin B2 and plexin B1 in HCAECs, which implies that Sema4D has multiple proinflammatory functions in ECs.

In this study, we revealed that KD sera can induce inflammatory cytokine production of HCAECs in a Sema4D-plexin-dependent manner. The effect of KD sera on endothelium inflammation has been recently reported [4, 37]. In their study, KD sera stimulated HCAECs to secret IL-8 and IL-1*β* by activating the Ca^2+^-nuclear factor of the activated T cell pathway and PPAR*γ*-JAK-STAT, respectively. Our findings are consistent with their views; that is, sera from KD patients act as an inducer of dysfunction and inflammation of ECs, leading to the overexpression of proinflammatory factors. Importantly, we found that both plexin B1 and plexin B2 were upregulated in HCAECs after KD serum stimulation, which enhanced the expression of endothelium proinflammatory cytokines. Indeed, Sema4D-plexin-mediated endothelium dysfunctions have been uncovered in diabetic retinopathy [19] and HBV infection [38]. Therefore, we can conclude that the Sema4D-plexin axis may be one of extensive proinflammatory signaling in inflammation status, or there may be a crosstalk between the Sema4D-plexin axis and other proinflammatory pathways.

There are some limitations in our study. First, we used CD15 as the surface marker of neutrophils in FCM, while eosinophil is also a kind of CD15^+^ granulocyte and constitutively expresses mSema4D [21]. Although the existing evidence shows that eosinophils, a small part of leukocytes, are not important cells in KD pathogenesis [39] and we did not find a relationship between sSema4D and peripheral eosinophil counts either in this study (data not shown), we cannot exclude eosinophil's contribution to the sSema4D elevation in KD. Second, it is well known that Sema4D is the main ligand of plexin B1, but a recent study showed that Sema3C can also activate plexin B1 [40]. We did not measure Sema3C concentration in the serum of KD patients, and whether Sema3C can also have a proinflammatory function in ECs is unclear. Last, due to the heterogeneity of serum composition, the smaller sample size of KD patient sera in this study might weaken our findings. In the future, we need further mechanism researches to evaluate the exact role of Sema4D in KD vasculitis.

In summary, our study showed that sSema4D level is increased in the serum of KD patients and is correlated with disease severity; in KD conditions, ADAM17-mediated mSema4D shedding from neutrophils contributes to sSema4D elevation; KD sera promote ECs to produce IL-1*β*, IL-6, and IL-8 by both the increased sSema4D and upregulating plexin B1 and plexin B2 expression on ECs. The findings demonstrated that the Sema4D-plexin B axis is responsible for the proinflammatory cytokine production of the endothelium in KD and is involved in the pathogenesis of KD-CAL. Sema4D may have potential as a new biomarker for the prediction of the KD-CAL degree and may provide a novel therapeutic target for KD treatment.

## Figures and Tables

**Table 1:** Comparison of demographic characteristics and sSema4D levels between KD patients and healthy controls.

Groups	Kawasaki disease	Healthy control	*p* value
Number	50	20	
Male	29	12	
Female	21	8	
Age (months)	32.07 ± 18.12	20.81 ± 18.86	0.180
sSema4D (pg/ml)	397.80 ± 18.02	242.70 ± 23.15	<0.001

Data are expressed as means ± standard deviation for normally distributed data or number for categorical variables.

## Data Availability

The data used to support the findings of this study are available from the corresponding author upon request.
